# A Técnica de Duplicação de Orifício na Plastia Valvar Mitral: 35 Anos de História

**DOI:** 10.36660/abc.20210067

**Published:** 2021-09-01

**Affiliations:** 1 Faculdade de Medicina da Universidade de São Paulo Hospital das Clínicas Instituto do Coração São Paulo SP Brasil Instituto do Coração do Hospital das Clínicas da Faculdade de Medicina da Universidade de São Paulo, São Paulo, SP - Brasil

**Keywords:** Valva Mitral/cirurgia, Doenças das Valvas Cardíacas/cirurgia, Morbidade e Mortalidade, Tromboembolismo/prevenção e controle, Endocardite/prevenção e controle

As vantagens da plastia valvar mitral são menor morbimortalidade, redução do risco de tromboembolismo e endocardite, melhora na sobrevida e melhor preservação da função ventricular esquerda. A causa mais comum de regurgitação mitral degenerativa é a válvula mixomatosa com prolapso segmentar do folheto posterior; esta lesão pode ser corrigida por técnicas clássicas, como a ressecção quadrangular, mas outras lesões podem exigir técnicas cirúrgicas mais complexas, por exemplo, correção de prolapso do folheto anterior ou doença de Barlow. A plastia valvar mitral em pacientes reumáticos ainda é um desafio.

**Figura 1:**
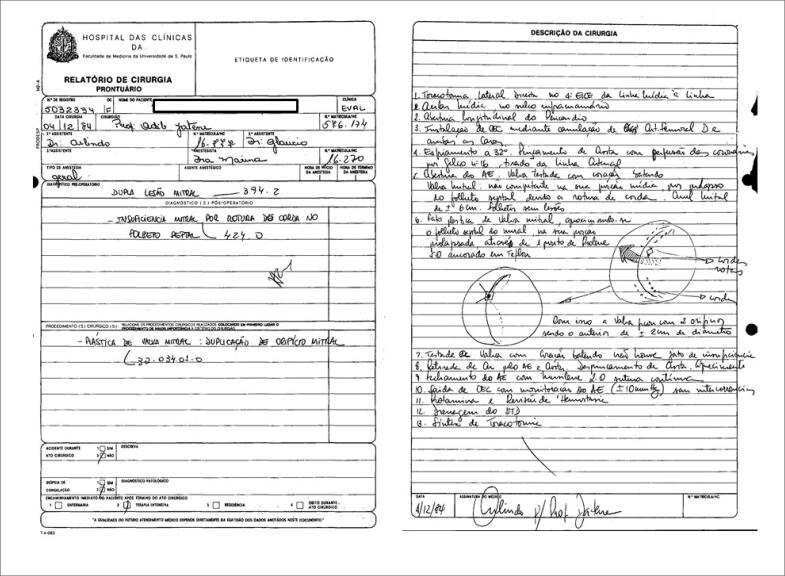
Relatório de cirurgia de plastia valvar mitral pela técnica de duplicação de orifício.

Em dezembro 1984, Adib Domingos Jatene MD, PhD realizou a primeira “duplicação de orifício mitral”, em uma paciente feminina com insuficiência mitral por ruptura das cordas do folheto anterior (Figura 1). A técnica restaurou a competência mitral ancorando a borda livre dos folhetos em prolapso à borda livre correspondente do folheto oposto com um ponto de polipropileno 5-0 reforçado com compressas. A paciente apresentou boa evolução imediata e tardia.

Em 1998, Maisano et al.^1^ publicaram a técnica de “duplicação de orifício mitral”, conhecida como a técnica “borda a borda”, que vinha sendo realizada desde 1991, segundo os autores. Subsequentemente, Alfieri et al. publicaram os resultados de médio prazo desta técnica única,^2^ mostrando a sua eficácia e durabilidade, e o mesmo grupo recentemente publicou os resultados de longo prazo (18 anos).^3^

Desde 1980, nós realizamos a plastia valvar mitral com diferentes técnicas, incluindo a “duplicação de orifício mitral”, conforme publicado em 1994 pelo nosso grupo,^4^ com bons resultados a longo prazo (17 anos).^5^ Realizamos este tipo de plastia valvar mitral em pacientes com anel valvar largo no sentido de não haver risco de causar estenose. Evidentemente, a degeneração dos tecidos das cúspides deve ser avaliada no ato operatório, assim como as cordas tendinosas e os músculos papilares, mas é necessário lembrar que, corrigida a insuficiência mitral, o estresse das cúspides é fortemente reduzido. Outro detalhe importante em qualquer plastia valvar mitral é a análise da coaptação entre as cúspides anterior e posterior, por meio do teste de solução salina, injetada pela mitral no ventrículo esquerdo. Quando utilizamos a duplicação de orifício mitral, devemos verificar não apenas a coaptação no local da duplicação, que obviamente é boa, visto que existe uma sutura entre as cúspides, mas também a coaptação em toda a extensão da borda entre as cúspides anterior e posterior.
