# Supplementary material for: A Técnica de Duplicação de Orifício na Plastia Valvar Mitral: 35 Anos de História
Source: Arq Bras Cardiol. 2021 Sep 1;117(3):424–5. [Article in Portuguese] doi: 10.36660/abc.20210067 (PMC8462944; doi:10.36660/abc.20210067)
Supplement: Translation

# Translation: The Double-Orifice Technique in Mitral Valve Repair: 35 Years of History

**Affiliations:** 1 São Paulo SP Brazil Instituto do Coração do Hospital das Clínicas da Faculdade de Medicina da Universidade de São Paulo, São Paulo, SP - Brazil

**Keywords:** Mitral Valve/surgery, Heart Valve Diseases/surgery, Morbidity and Mortality, Thromboembolism/prevention and control, Endocarditis/prevention and control

The advantages of mitral valve repair are lower morbidity and mortality, reduced risk of thromboembolism and endocarditis, improved survival, and better preservation of left ventricular function. The most common cause of degenerative mitral regurgitation is a myxomatous valve with segmental prolapse of the posterior leaflet, a lesion that can be corrected by classical techniques such as quadrangular resection, but other lesions may require more complex surgical techniques, for instance, correction of anterior leaflet prolapse or Barlow disease. Mitral valve repair in rheumatic patients is still a challenge.

In December 1984, Adib Domingos Jatene MD, PhD performed the first “duplication of the mitral orifice”, in a female patient with mitral insufficiency due to rupture of anterior leaflet chordae (Figure 1). The technique restored mitral competence by anchoring the free edge of the prolapsing leaflets to the corresponding free edge of the opposing leaflet with a 5-0 polypropylene stitch reinforced with pledgets. The patient presented good immediate and long-term evolution.

**Figure 1:** Surgical report of mitral valve repair with the double-orifice technique.

In 1998, Maisano et al.,^1^ published the technique of “duplication of the mitral orifice”, known as the “edge-to-edge” technique, which had been performed since 1991 according to the authors. Subsequently, Alfieri et al. published the midterm results of this single technique,^2^ showing effectiveness and durability, and the same group recently published the long-term (18 years) results.^3^

Since 1980, we have been performing mitral valve repair with different techniques, including the “duplication of the mitral orifice”, as published in 1994 by our group,^4^ with good long-term (17 years) results.^5^ We perform this type of mitral valve repair in patients who have a large valve ring so that there is no risk of causing stenosis. Evidently, cusp tissue degeneration must be evaluated in the operative act, as well as the tendinous cords and the papillary muscles, but we must remember that once mitral insufficiency has been corrected, cusp stress is greatly reduced. Another important detail in any mitral valve repair is the analysis of coaptation between the anterior and posterior cusps, using the saline test, injected through the mitral into the left ventricle. When we use the mitral duplication of the mitral orifice, we must verify not only the coaptation in the duplication site, which is obviously good, given that there is a suture between the cusps, but also coaptation throughout the extent of the edge between the anterior and posterior cusps.
